# A Highly Potent Apomorphine Derivative Enhancing Neurite Outgrowth via Nrf2 Activation

**DOI:** 10.3390/antiox14050537

**Published:** 2025-04-29

**Authors:** Tamaki Ishima, Hitoshi Osaka, Ryozo Nagai, Kenichi Aizawa

**Affiliations:** 1 Division of Clinical Pharmacology, Department of Pharmacology, Jichi Medical University, Shimotsuke 329-0498, Japan; 2 Department of Pediatrics, Jichi Medical University, Shimotsuke 329-0498, Japan; 3 Jichi Medical University, Shimotsuke 329-0498, Japan; 4 Clinical Pharmacology Center, Jichi Medical University Hospital, Shimotsuke 329-0498, Japan; 5 Division of Translational Research, Clinical Research Center, Jichi Medical University Hospital, Shimotsuke 329-0498, Japan

**Keywords:** apomorphine derivatives, neurite outgrowth, edaravone, neurodegenerative diseases, therapeutic agents, Nrf2 activation

## Abstract

Apomorphine (APO), a dopamine agonist, activates nuclear factor erythroid 2-related factor 2 (Nrf2) and exerts antioxidant effects, making it a promising candidate for neuroprotection against oxidative stress. This study evaluated neuroplasticity-enhancing properties of newly synthesized APO derivatives, focusing on their ability to promote neurite outgrowth in PC12 cells under nerve growth factor (NGF) stimulation. D55, an APO derivative, retains the hydroxyl group at APO’s 11th position while substituting the 10th with an ethoxy group. D55 exhibited the highest potency (EC_50_ = 0.5661 nM), significantly enhancing neurite outgrowth. APO demonstrated the highest efficacy (Emax ~10-fold increase), while edaravone (Eda) required higher concentrations (EC_50_ = 22.5 nM) for moderate effects (Emax ~4-fold increase). D30, in which the 11th hydroxyl was replaced with a methoxy group, had no effect. Neurite outgrowth-promoting effects of APO, D55, and Eda were significantly attenuated by Nrf2 siRNA knockdown, confirming that their neuroplasticity effects are Nrf2-mediated. These findings confirm that D55 is a highly potent Nrf2-activating compound with strong neuroprotective potential, providing new insights into its therapeutic applications for neurodegenerative diseases associated with oxidative stress.

## 1. Introduction

**Figure 1:**
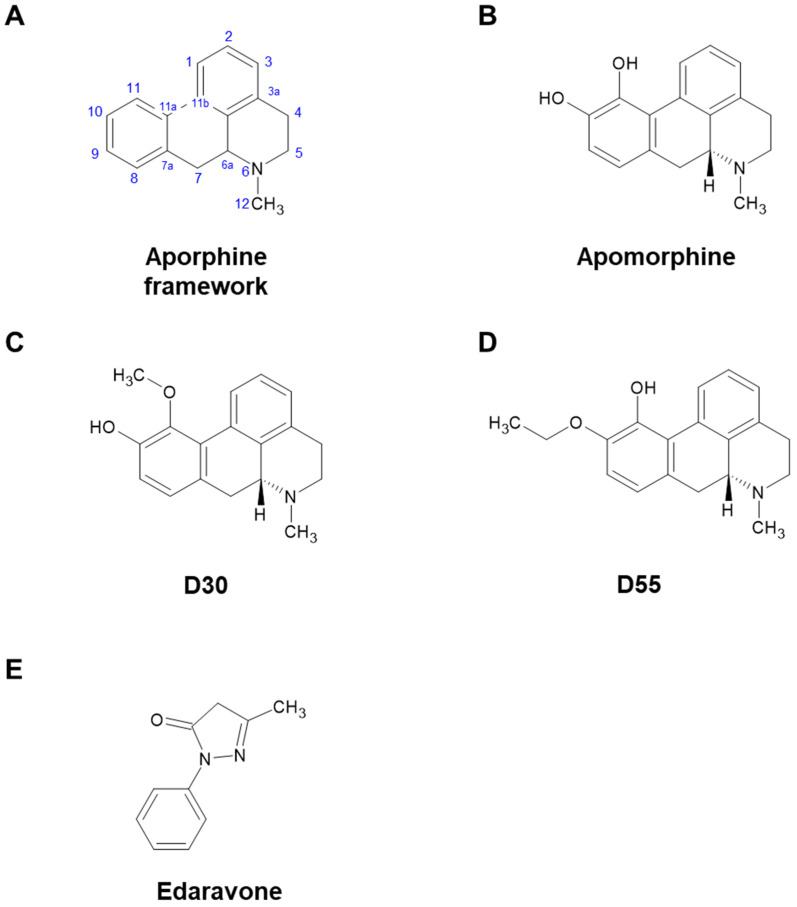
Chemical structures of apomorphine, D30, D55, and edaravone. (**A**) Aporphine framework. (**B**) Apomorphine (APO) has two hydroxy groups at positions 10 and 11. (**C**) D30 possesses a hydroxy group at position 10 and a methoxy group at position 11 in the aporphine alkaloid framework. (**D**) In D55, an ethoxy group occupies position 10, with a hydroxy group at position 11 in the aporphine alkaloid framework. (**E**) Edaravone (Eda) is a free radical scavenger, but its structure does not contain an aporphine alkaloid framework.

Apomorphine (APO), a drug used to treat Parkinson’s disease and other disorders, is primarily known as a dopaminergic (DA) agonist. However, it also possesses many other potentially useful properties [1,2]. Miyauchi et al. identified APO as an agent that rescues apoptosis of fibroblasts induced by reactive oxygen species in pediatric patients with mitochondrial encephalomyopathies [3]. However, in order to reposition APO as a therapeutic agent for mitochondrial diseases, the strong DA effect leading to emesis, an undesirable side effect, became a challenge. Therefore, Kobayashi et al. synthesized 20 derivatives with an aporphine framework (Figure 1A) and screened their DA activity and anti-cell death effect under oxidative stress. As a result, from the parent compound, APO (Figure 1B, IUPAC: 6-Methyl-5,6,6a,7-tetrahydro-4H dibenzo[de,g]quinoline-10,11-diol), we obtained D30 (Figure 1C, IUPAC: 11-Methoxy-6-methyl-5,6,6a,7-tetrahydro-4H dibenzo[de,g]quinolin-10-ol) and D55 (Figure 1D, IUPAC: 10-Ethoxy-6-methyl-5,6,6a,7-tetrahydro-4H dibenzo[de,g]quinolin-11-ol). D30 lost its anti-cell death effect, but D55 showed better anti-cell death effect (EC_50_ = 4 nM) than APO [4]. It has been suggested that the anti-cell death effect of APO involves the nuclear factor erythroid 2-related like 2 (Nrf2) Antioxidant Response Element (ARE) pathway [5]. Recently, in a study using a model of steatohepatitis, Maeda et al. reported that APO activates Nrf2 and inhibits ferroptosis [6]. In studies of ischemic stroke and ALS models, edaravone (Eda) (Figure 1E), a cerebroprotective agent, was also approved for treatment of amyotrophic lateral sclerosis (ALS) and has been reported as an Nrf2 activator [7,8,9].

It has been reported that Nrf2 is involved in neuroprotection, including neuroplasticity [10]. The PC12 cell line, derived from rat adrenal medullary pheochromocytoma, has been used to study neuronal plasticity because it differentiates into neuron-like cells and promotes neurite outgrowth in response to drugs such as nerve growth factor (NGF) [11,12,13]. However, in studies involving APO, there are only reports of its use to measure cytotoxicity in Parkinsonian models [14], and to our knowledge, there are no reports of its effects on neuroplasticity. In this study, we first determined whether these compounds induce NGF-mediated neurite outgrowth in PC12 cells and then calculated their potency. Furthermore, the role of Nrf2 in neuroplasticity was verified through RNAi experiments.

## 2. Materials and Methods

### 2.1. Compounds

Compounds were obtained from the following sources: (R)-(-)-Apomorphine Hydrochloride Hemihydrate was sourced from Fujifilm Wako Pure Chemicals Corporation (#013-18323, Osaka, Japan), and 3-Methyl-1-phenyl-5-pyrazolone (Eda) from Tokyo Chemical Industry Co., Ltd. (#M0687, Tokyo, Japan). D30 (CAS No. 36507-69-4) and D55 (CAS No. 2926569-76-6) were derived from apomorphine as the parent compound [4].

### 2.2. Quantification of Neurite Outgrowth in PC12 Cells

PC12 cells (RIKEN Cell Bank, Tsukuba, Japan) were cultured at 37 °C and 5% CO_2_ in Dulbecco’s Modified Eagle’s Medium (DMEM), supplemented with 10% heat-inactivated fetal bovine serum (FBS), 5% heat-inactivated horse serum, and 1% Gibco™ penicillin–streptomycin (10,000 U/mL: Thermo Fisher Scientific, Waltham, MA, USA). The medium was changed two to three times a week. PC12 cells were plated onto 24-well tissue culture plates coated with poly-D-lysine/laminin. Cells were plated at a relatively low density (0.25 × 10^4^ cells/cm^2^) in DMEM medium containing 0.5% FBS and 1% penicillin–streptomycin. Medium containing a minimal level of serum (0.5% FBS) was used as previously reported [15,16,17]. Twenty-four hours after plating, the medium was replaced with DMEM medium containing 0.5% FBS and 1% penicillin–streptomycin with NGF (2.5 ng/mL), with or without 0.001, 0.01, 0.1, 1, or 10 μM of each compound.

Four days after incubation with NGF (2.5 ng/mL) with or without the specified compound, morphometric analysis was performed on digitized images of live cells taken under phase contrast illumination using an inverted microscope linked to a camera. An average of 100 cells per field was analyzed. Differentiated cells were counted by visual examination of the field. Only cells with at least one neurite were counted and expressed as a percentage of all cells in the field. Counting was performed in a blinded manner. Data were expressed as a percentage of the control group (NGF alone).

RNAi gene expression knockdown studies were performed according to previously reported methods [18,19], using the following sequence: *Rattus norvegicus* Nrf2 (*Nfe2l2*), mRNA GenBank Accession No. NM_031789: sense, 5′-GCCAAAGCUAGUGUAGAAAAUAUAT-3′; antisense, 5′-AUAUUAUUCUACACUAGCUUGGCUA-3′. For all relative control experiments, cells were exposed to scrambled non-specific RNAi duplex with the following sequence: sense, 5′-CUCCUUCUCUCCCUUGUGA-3′; antisense, 5′-UCACAAGGGAGAAAGAGAGGAAGGA-3′. Each RNAi duplex was synthesized as chimeras, incorporating two DNA nucleotides in the 25-mer sense strand to enhance RNAi efficacy. These were then annealed with the 27-mer antisense strand (FASMAC Co., Ltd., Atsugi, Japan) and introduced into cells using Lipofectamine 2000 reagent (Invitrogen, Carlsbad, CA, USA) following the manufacturer’s guidelines.

### 2.3. Statistical Analysis

Statistical analysis was performed using GraphPad Prism (version 7.00, GraphPad Software, Boston, MA, USA). Data were analyzed by one-way analysis of variance (ANOVA) or two-way ANOVA. As appropriate, post hoc comparisons were performed using the Bonferroni test or Tukey’s Honestly Significant Difference test. Data are presented as means ± standard errors of the mean (S.E.M.), with statistical significance set at *p* < 0.05.

## 3. Results

### 3.1. Effects of APO, Its Derivatives, and Eda on NGF-Induced Neurite Outgrowth in PC12 Cells

Effects of each compound on NGF-induced neurite outgrowth in PC12 cells were examined. DMEM medium containing the minimum amount of FBS (0.5%) in which cells could survive and 2.5 ng/mL NGF, the minimum requirement for neuronal differentiation, was used as a control.

**Figure 2:**
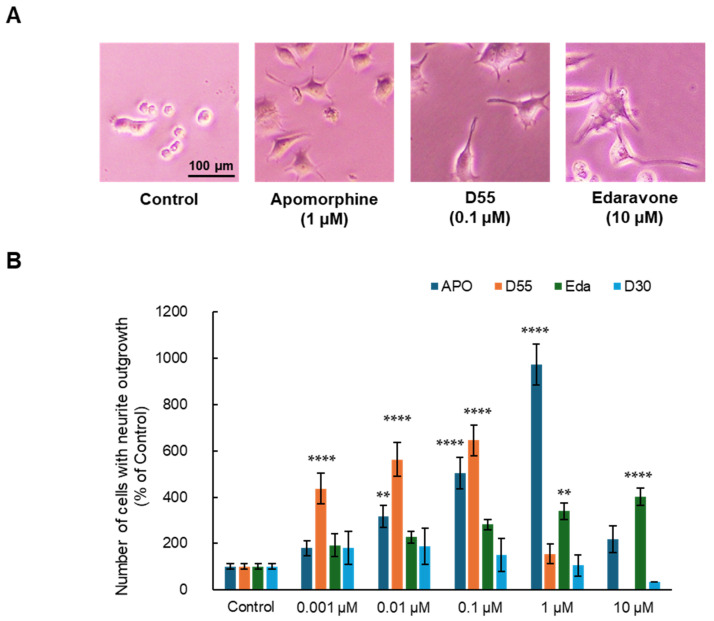
Potentiation of neurite outgrowth by apomorphine, D55, and edaravone. (**A**) Representative photographs in PC12 cells. Control: NGF (2.5 ng/mL) alone, apomorphine, D55, and edaravone: NGF (2.5 ng/mL) + APO (1 µM), D55 (0.1 µM), or Eda (10 µM). (**B**) Effects of APO and derivatives on NGF-induced neurite outgrowth in PC12 cells. APO (0.001, 0.01, 0.1, and 1 µM), D55 (0.001, 0.01, and 0.1 µM), and Eda (0.001, 0.01, 0.1, 1, and 10 µM) potentiated neurite outgrowth in PC12 cells, in a concentration-dependent manner. All data represent the mean ± S.E.M. (n = 7–8). ** *p* < 0.01, **** *p* < 0.0001 as compared with the control group (one-way ANOVA).

As a result, APO, D55, and Eda exhibited neurite outgrowth effects at concentrations of 1 µM, 0.1 µM, and 10 µM, respectively (Figure 2A). APO, D55, and Eda exhibited concentration-dependent neurite outgrowth effects at concentrations up to 1 µM, 0.1 µM, and 10 µM, respectively, while D30 had no effect (Figure 2B). APO exhibited an Emax of approximately 10-fold neurite outgrowth compared to the control at 1 µM, whereas D55 exhibited an Emax of about 4-fold neurite outgrowth compared to the control at concentrations as low as 1 nM. Eda showed no toxicity at concentrations as high as 10 µM.

### 3.2. EC_50_ of APO, Its Derivatives, and Eda

**Figure 3:**
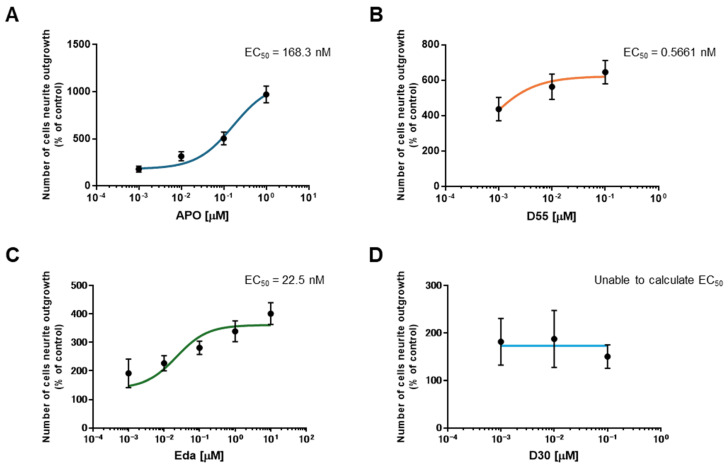
EC_50_s for each Compound. EC_50_s were calculated for (**A**) APO, (**B**) D55, and (**C**) Eda. The EC_50_ for (**D**) D30 could not be calculated because it did not promote neurite outgrowth. Control: NGF (2.5 ng/mL) alone.

To evaluate the potency of each drug, the half-maximal effective concentration (EC_50_) was calculated. EC_50_ values for APO, D55, and Eda were 168.3 nM, 0.5661 nM, and 22.5 nM, respectively (Figure 3A–C). For D30, the EC_50_ could not be calculated as it does not promote neurite outgrowth (Figure 3D).

### 3.3. Role of Nrf2 in Potentiating NGF-Induced Neurite Outgrowth by APO, D55, and Eda

**Figure 4:**
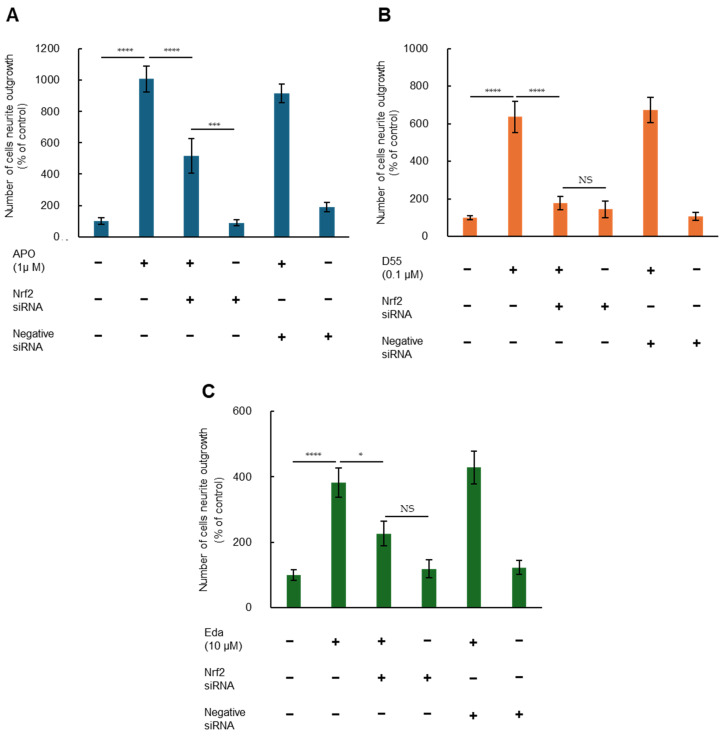
Nrf2 enhances nerve growth factor (NGF)-induced neurite outgrowth by APO, D55, and Eda. Potentiating effects of (**A**) APO (1 µM), (**B**) D55 (0.1 µM), or (**C**) Eda (10 µM) on NGF-induced neurite outgrowth were significantly antagonized by treatment with Nrf2 siRNA, but not negative siRNA. Neither Nrf2 siRNA nor negative siRNA alone altered NGF (2.5 ng/mL)-induced neurite outgrowth. All data represent the mean ± S.E.M. (n = 8). * *p* < 0.05, *** *p* < 0.001, **** *p* < 0.0001 as compared with the APO (1 µM), D55 (0.1 µM), or Eda (10 µM) group (two-way ANOVA). NS = not significant, *p* > 0.05.

To investigate whether Nrf2 is involved in potentiation of NGF-induced neurite outgrowth by APO, D55, and Eda, we examined effects of Nrf2 gene knockdown. Two-way ANOVA of neurite outgrowth data showed significant effects, indicating that potentiation effects of APO (1.0 µM), D55 (0.1 µM), and Eda (10 µM) on NGF-induced neurite outgrowth were significantly attenuated by treatment with Nrf2 siRNA, but not by the negative control (Figure 4). In contrast, treatment with either Nrf2 siRNA or the negative control alone did not alter NGF-induced neurite outgrowth in PC12 cells (Figure 4). These findings suggest that APO, D55, and Eda enhance NGF-induced neurite outgrowth through activation of Nrf2. However, in the case of APO, attenuation of the neurite outgrowth effect by Nrf2 siRNA treatment was partial (Figure 4A).

## 4. Discussion

### 4.1. Neuroprotective Effect of APO

A previous study by Miyauchi et al. screened apomorphine (APO) as a candidate drug for pediatric mitochondrial disease [3]. APO is a drug to improve off symptoms of Parkinson’s disease that reportedly has neuroprotective properties as well [20,21,22,23]. Recently, its neuroprotective effects due to its antioxidant properties [24], its anti-ferroptosis effects on fibroblasts [25], and its ability to reduce fatty liver and inflammation in a metabolic dysfunction-associated steatohepatitis (MASH) animal model [26] have been reported. It is thought to act as a radical scavenger [27] through activation of the Nrf2-ARE pathway [5].

### 4.2. Cell-Based In Vitro Model Using PC12 Cells

The PC12 pheochromocytoma cell line, derived from rat adrenal tumors, was established in 1976 [28]. Treatment of these cells with NGF halts their proliferation and promotes neurite outgrowth. Although not neurons, they have been used as in vitro neuronal-like models for studies of NGF signaling and neuronal differentiation [29,30,31], as well as models of cytotoxicity, including serum deprivation [32], toxic molecules [33], and neurodegenerative diseases [34,35]. Additionally, they are often used as in vitro models to identify neuroprotective agents. In this study, we investigated neurite outgrowth effects of APO and its derivatives, as well as Eda [36], which was used as a positive control for its radical scavenger properties.

As a result, concentration-dependent neurite outgrowth effects were observed with APO, its derivative D55, and Eda. APO has hydroxyl groups at positions 10 and 11 of the aporphine framework (Figure 1A,B). D30, which has a methoxy group at position 11 and lacks an anti-cell death effect, did not exhibit neurite outgrowth (Figure 1C). D55, which retains the hydroxyl group at position 11 (considered to have anti-cell death effects) and possesses an ethoxy group at position 10, showed similar effects (Figure 1D) [4]. Preliminary studies indicated that Eda did not induce cell death in PC12 cells even at high concentrations (100 µM). However, in this study, APO induced partial cell death at 10 µM, as did D55 at concentrations above 1 µM. At 1 µM, APO exhibited an Emax about ten-fold more effective than the control in promoting neurite outgrowth, whereas D55 exhibited an Emax four-fold more effective than the control, even at a concentration of 1 nM.

The EC_50_ of each drug was calculated, with D55 having the lowest value at 0.5661 nM. APO causes neurotoxicity and neurodegeneration in animal models at high concentrations [37,38]. It also induces apoptosis in choriocarcinoma at concentrations of 10 µM or higher [39]. D55, which is potent at low concentrations, may have an advantage over APO and Eda in terms of reducing the risk of adverse effects.

### 4.3. Neurite Outgrowth and Nrf2

It has already been reported that the phytochemical, sulforaphane, in broccoli sprouts [18], Astragaloside IV in dried roots of *Astragalus membranaceus* (Radix Astragali) [40], and Nrf2 activators, such as TBE-31 and MCE-1 [19], enhance neurite outgrowth via Nrf2-mediated pathways. Although there are reports that both APO and Eda activate Nrf2 [6,41,42], there have been no reports on their effects on neurite outgrowth. In the present study, we sought to determine whether Nrf2 is associated with neurite outgrowth in PC12 cells and whether this is also the case for D55. Potentiating effects of APO, D55, or Eda on NGF-induced neurite outgrowth were significantly antagonized by Nrf2 siRNA treatment, but not by negative siRNA. Nrf2 siRNA and negative siRNA alone did not alter NGF-induced neurite outgrowth. Thus, these neurite outgrowth effects proved to be associated with Nrf2 expression.

Regarding APO, inhibition of neurite outgrowth effects by Nrf2 siRNA was partial (Figure 4A). Studies on atypical antipsychotic drugs, aripiprazole and brexpiprazole, have reported that the selective 5-hydroxytryptamine (5-HT)_1A_ receptor antagonist, WAY100635, inhibits neurite outgrowth, whereas the agonist 8OH-DPAT has neurite outgrowth-promoting effects. Additionally, the selective 5-HT_2A_ receptor agonist, DOI, does not have neurite outgrowth-promoting effects, whereas the antagonist, M100907, promotes neurite outgrowth [16,17]. Since APO acts as a partial agonist of 5-HT_1A_ receptors and an antagonist of 5-HT_2A_ receptors [1,43], it is possible that some effects not inhibited by Nrf2 siRNA are related to serotonin receptor activity.

### 4.4. Neuroplasticity

There are many reports that Nrf2 is involved in neuroprotection [44]; however, in cellular models, the only reports on Nrf2 activation so far are related to neuroprotection under oxidative stress or against cytotoxicity, as shown in cell viability studies [45]. Even in studies limited to PC12 cells, research with APO has only reported neuroprotection against MPTP and 6-hydroxydopamine [46]. Neurite outgrowth observed with PC12 cells in the present study demonstrated the neurite outgrowth effect of the drug itself, independent of neuroprotection. These findings indicate that APO, D55, and Eda are associated with neuroplasticity. This is the first report on neuroplasticity of APO and its new derivatives, D55 and Eda.

Nrf2 is a modulator of proteostasis and has been implicated in Parkinson’s disease [47,48] and other neurodegenerative diseases [49] such as Alzheimer’s disease [50,51], Huntington’s disease [52,53], ALS [54,55], and ataxia [56,57]. It is also associated with psychiatric disorders such as depression [58,59] and schizophrenia [60,61], as well as stroke [62,63], traumatic brain injury [64,65], and multiple sclerosis [66,67]. D55, an Nrf2 activator that is unlikely to have DA adverse effects, appears to be a promising compound for treating neurological and psychiatric disorders, in addition to mitochondrial encephalomyopathies, due to its neuroprotective and neuroplasticity effects.

### 4.5. Limitations

The primary limitation of this study is that it was conducted solely using PC12 cells. Since Nrf2 is essential not only in oxidative stress, but also in proteostasis, detailed analyses need to be conducted using KO mice and other models. Additionally, further investigation is required to understand the effects of D55 beyond the D2 receptor. Detailed mechanisms of neuroplasticity remain unknown. According to Tebey et al., an excess of Nrf2 may disrupt the balance of homeostasis toward by causing reductive stress, which promotes protein misfolding [68]. It is believed that low levels of Nrf2 in the brain require only slight upregulation to be beneficial under pathological conditions [49]. Despite challenges in practical applications, we think that drugs like D55, which promote neurite outgrowth at low concentrations, may be advantageous in this regard.

Furthermore, in the future, we plan to (i) investigate other signaling pathways involved in neurite outgrowth, beyond Nrf2, (ii) perform long-term viability studies and LD_50_ assessments to evaluate safety margins of these compounds, (iii) assess additional neurite outgrowth markers such as β-III tubulin, GAP-43, and PSD-95, and (iv) test other neurotrophic factors and Nrf2 activators/inhibitors beyond NGF, to further validate neuroplasticity-enhancing effects of these compounds.

## 5. Conclusions

D55 exhibits NGF-induced neurite outgrowth effects in PC12 cells and is more effective at lower concentrations than the parent compound, APO, or the free radical scavenger, Eda. These results indicate that D55 not only prevents cell death but also has neuroplastic properties. We confirmed that this effect is mediated by Nrf2. Drugs that promote Nrf2-mediated neuroplasticity without the side effects of D55 are expected to be useful in the treatment of neurological and psychiatric disorders.

## Data Availability

All data are included within the manuscript.

## Abbreviations

The following abbreviations are used in this manuscript:

APO	Apomorphine
Nrf2	nuclear factor erythroid 2-related factor 2
Eda	Edaravone
DA	dopaminergic
ARE	Antioxidant Response Element
ALS	Amyotrophic lateral sclerosis
NGF	Nerve Growth Factor
MASH	Metabolic dysfunction steatohepatitis
5-HT	5-hydroxytryptamine
MPTP	1-methyl-4-phenyl-1,2,3,6-tetrahydropyridine

## References

[1] Auffret M., Drapier S., Vérin M. (2018). Pharmacological Insights into the Use of Apomorphine in Parkinson’s Disease: Clinical Relevance. Clin. Drug Investig..

[2] Ribarič S. (2012). The pharmacological properties and therapeutic use of apomorphine. Molecules.

[3] Miyauchi A., Kouga T., Jimbo E.F., Matsuhashi T., Abe T., Yamagata T., Osaka H. (2019). Apomorphine rescues reactive oxygen species-induced apoptosis of fibroblasts with mitochondrial disease. Mitochondrion.

[4] Kobayashi M., Miyauchi A., Jimbo E.F., Oishi N., Aoki S., Watanabe M., Yoshikawa Y., Akiyama Y., Yamagata T., Osaka H. (2024). Synthetic aporphine alkaloids are potential therapeutics for Leigh syndrome. Sci. Rep..

[5] Hara H., Ohta M., Adachi T. (2006). Apomorphine protects against 6-hydroxydopamine-induced neuronal cell death through activation of the Nrf2-ARE pathway. J. Neurosci. Res..

[6] Maeda H., Miura K., Aizawa K., Bat-Erdene O., Sashikawa-Kimura M., Noguchi E., Watanabe M., Yamada N., Osaka H., Morimoto N. (2024). Apomorphine Suppresses the Progression of Steatohepatitis by Inhibiting Ferroptosis. Antioxidants.

[7] Barati A., Moghimi S., Taghavi Zanjani K., Rohani M., Sohrabi Hesar M., Arfaie A., Ghezelche Khamsiyan M., Mahmoudi J., Sadigh-Eteghad S. (2025). Acute Administration of Edaravone Improves Cognitive Impairment in a Mouse Model of mPFC Ischemia: Crosstalk Between Necroptosis, Neuroinflammation, and Antioxidant Defense. Mol. Neurobiol..

[8] Bajpai A., Bharathi V., Patel B.K. (2025). Therapeutic targeting of the oxidative stress generated by pathological molecular pathways in the neurodegenerative diseases, ALS and Huntington’s. Eur. J. Pharmacol..

[9] Soejima-Kusunoki A., Okada K., Saito R., Watabe K. (2022). The Protective Effect of Edaravone on TDP-43 Plus Oxidative Stress-Induced Neurotoxicity in Neuronal Cells: Analysis of Its Neuroprotective Mechanisms Using RNA Sequencing. Pharmaceuticals.

[10] Hashimoto K. (2018). Essential Role of Keap1-Nrf2 Signaling in Mood Disorders: Overview and Future Perspective. Front. Pharmacol..

[11] Meng P., Zhu L., Guo J., Li Y., Wei Y., Sun J., Zhu J. (2024). Preparation of recombinant neuritin protein. Protein Expr. Purif..

[12] Wang F., Wang H., Shan X., Mei J., Wei P., Song Q., Chen W. (2023). High-strength and high-toughness ECM films with the potential for peripheral nerve repair. Biomed. Mater..

[13] Johansen V.B.I., Hampson E., Tsonou E., Pantarelli C., Chu J.Y., Crossland L., Okkenhaug H., Massey A.J., Hornigold D.C., Welch H.C.E. (2023). The GPCR adaptor protein Norbin regulates S1PR1 trafficking and the morphology, cell cycle and survival of PC12 cells. Sci. Rep..

[14] Gassen M., Gross A., Youdim M.B. (1998). Apomorphine enantiomers protect cultured pheochromocytoma (PC12) cells from oxidative stress induced by H2O2 and 6-hydroxydopamine. Mov. Disord. Off. J. Mov. Disord. Soc..

[15] Hashimoto K., Ishima T. (2010). A novel target of action of minocycline in NGF-induced neurite outgrowth in PC12 cells: Translation initiation [corrected] factor eIF4AI. PLoS ONE.

[16] Ishima T., Iyo M., Hashimoto K. (2012). Neurite outgrowth mediated by the heat shock protein Hsp90α: A novel target for the antipsychotic drug aripiprazole. Transl. Psychiatry.

[17] Ishima T., Futamura T., Ohgi Y., Yoshimi N., Kikuchi T., Hashimoto K. (2015). Potentiation of neurite outgrowth by brexpiprazole, a novel serotonin-dopamine activity modulator: A role for serotonin 5-HT1A and 5-HT2A receptors. Eur. Neuropsychopharmacol. J. Eur. Coll. Neuropsychopharmacol..

[18] Yao W., Zhang J.C., Ishima T., Dong C., Yang C., Ren Q., Ma M., Han M., Wu J., Suganuma H. (2016). Role of Keap1-Nrf2 signaling in depression and dietary intake of glucoraphanin confers stress resilience in mice. Sci. Rep..

[19] Yao W., Zhang J.C., Ishima T., Ren Q., Yang C., Dong C., Ma M., Saito A., Honda T., Hashimoto K. (2016). Antidepressant effects of TBE-31 and MCE-1, the novel Nrf2 activators, in an inflammation model of depression. Eur. J. Pharmacol..

[20] Yuan H., Sarre S., Ebinger G., Michotte Y. (2004). Neuroprotective and neurotrophic effect of apomorphine in the striatal 6-OHDA-lesion rat model of Parkinson’s disease. Brain Res..

[21] Fornai F., Battaglia G., Gesi M., Orzi F., Nicoletti F., Ruggieri S. (2001). Dose-dependent protective effects of apomorphine against methamphetamine-induced nigrostriatal damage. Brain Res..

[22] Castri P., Busceti C., Battaglia G., Girardi F., Cavallari M., Orzi F., Fornai F. (2006). Protection by apomorphine in two independent models of acute inhibition of oxidative metabolism in rodents. Clin. Exp. Hypertens..

[23] Hara H., Maeda A., Kamiya T., Adachi T. (2013). Protective effects of apomorphine against zinc-induced neurotoxicity in cultured cortical neurons. Biol. Pharm. Bull..

[24] Ikram H., Zakir R., Haleem D.J. (2024). Memory enhancing and neuroprotective effects of apomorphine in a rat model of dementia. Metab. Brain Dis..

[25] Miyauchi A., Watanabe C., Yamada N., Jimbo E.F., Kobayashi M., Ohishi N., Nagayoshi A., Aoki S., Kishita Y., Ohtake A. (2024). Apomorphine is a potent inhibitor of ferroptosis independent of dopaminergic receptors. Sci. Rep..

[26] Ogiso H., Miura K., Nagai R., Osaka H., Aizawa K. (2024). Non-Targeted Metabolomics Reveal Apomorphine’s Therapeutic Effects and Lysophospholipid Alterations in Steatohepatitis. Antioxidants.

[27] Gassen M., Glinka Y., Pinchasi B., Youdim M.B. (1996). Apomorphine is a highly potent free radical scavenger in rat brain mitochondrial fraction. Eur. J. Pharmacol..

[28] Greene L.A., Tischler A.S. (1976). Establishment of a noradrenergic clonal line of rat adrenal pheochromocytoma cells which respond to nerve growth factor. Proc. Natl. Acad. Sci. USA..

[29] Zachor D.A., Moore J.F., Brezausek C.M., Theibert A.B., Percy A.K. (2000). Cocaine inhibition of neuronal differentiation in NGF-induced PC12 cells is independent of ras signaling. Int. J. Dev. Neurosci. Off. J. Int. Soc. Dev. Neurosci..

[30] Xiong A., Yan A.L., Bi C.W., Lam K.Y., Chan G.K., Lau K.K., Dong T.T., Lin H., Yang L., Wang Z. (2016). Clivorine, an otonecine pyrrolizidine alkaloid from Ligularia species, impairs neuronal differentiation via NGF-induced signaling pathway in cultured PC12 cells. Phytomed. Int. J. Phytother. Phytopharm..

[31] Yu C.L., Zhao X.M., Niu Y.C. (2016). Ferulic Acid Protects Against Lead Acetate-Induced Inhibition of Neurite Outgrowth by Upregulating HO-1 in PC12 Cells: Involvement of ERK1/2-Nrf2 Pathway. Mol. Neurobiol..

[32] Greene L.A. (1978). Nerve growth factor prevents the death and stimulates the neuronal differentiation of clonal PC12 pheochromocytoma cells in serum-free medium. J. Cell Biol..

[33] Gassen M., Pergande G., Youdim M.B. (1998). Antioxidant properties of the triaminopyridine, flupirtine. Biochem. Pharmacol..

[34] Wei H., Leeds P.R., Qian Y., Wei W., Chen R., Chuang D. (2000). beta-amyloid peptide-induced death of PC 12 cells and cerebellar granule cell neurons is inhibited by long-term lithium treatment. Eur. J. Pharmacol..

[35] Matsumoto G., Stojanovic A., Holmberg C.I., Kim S., Morimoto R.I. (2005). Structural properties and neuronal toxicity of am-yotrophic lateral sclerosis-associated Cu/Zn superoxide dismutase 1 aggregates. J. Cell Biol..

[36] Homma T., Kobayashi S., Sato H., Fujii J. (2019). Edaravone, a free radical scavenger, protects against ferroptotic cell death in vitro. Exp. Cell Res..

[37] Picada J.N., Roesler R., Henriques J.A. (2005). Genotoxic, neurotoxic and neuroprotective activities of apomorphine and its oxidized derivative 8-oxo-apomorphine. Braz. J. Med. Biol. Res..

[38] Russo E., De Fazio S., De Fazio P., Amorosi A., Perrotta I., De Sarro G., Donato G. (2007). Apomorphine-induced neurodegen-eration in Mongolian gerbil hippocampus. Schizophr. Res..

[39] Lee J.Y., Ham J., Lim W., Song G. (2020). Apomorphine induces mitochondrial-dysfunction-dependent apoptosis in choriocarcinoma. Reproduction.

[40] Yu C., Pan S., Dong M., Niu Y. (2017). Astragaloside IV attenuates lead acetate-induced inhibition of neurite outgrowth through activation of Akt-dependent Nrf2 pathway in vitro. Biochim. Biophys. Acta Mol. Basis Dis..

[41] Yoshikawa N., Hirata N., Kurone Y., Shimoeda S. (2025). Edaravone is a Therapeutic Candidate for Doxorubicin-Induced Cardiomyopathy by Activating the Nrf2 Pathway. Pharmacol. Res. Perspect..

[42] Dang R., Wang M., Li X., Wang H., Liu L., Wu Q., Zhao J., Ji P., Zhong L., Licinio J. (2022). Edaravone ameliorates de-pressive and anxiety-like behaviors via Sirt1/Nrf2/HO-1/Gpx4 pathway. J. Neuroinflamm..

[43] Newman-Tancredi A., Cussac D., Quentric Y., Touzard M., Verrièle L., Carpentier N., Millan M.J. (2002). Differential actions of antiparkinson agents at multiple classes of monoaminergic receptor. III. Agonist and antagonist properties at serotonin, 5-HT(1) and 5-HT(2), receptor subtypes. J. Pharmacol. Exp. Ther..

[44] Sethi P., Mehan S., Khan Z., Maurya P.K., Kumar N., Kumar A., Tiwari A., Sharma T., Das Gupta G., Narula A.S. (2025). The SIRT-1/Nrf2/HO-1 axis: Guardians of neuronal health in neurological disorders. Behav. Brain Res..

[45] Lee H.R., Jee H.J., Jung Y.S. (2025). Neuroprotective Effect of β-Lapachone against Glutamate-Induced Injury in HT22 Cells. Biomol. Ther..

[46] Grünblatt E., Mandel S., Gassen M., Youdim M.B. (1999). Potent neuroprotective and antioxidant activity of apomorphine in MPTP and 6-hydroxydopamine induced neurotoxicity. J. Neural Transm. Suppl..

[47] Kim S., Indu Viswanath A.N., Park J.H., Lee H.E., Park A.Y., Choi J.W., Kim H.J., Londhe A.M., Jang B.K., Lee J. (2020). Nrf2 activator via interference of Nrf2-Keap1 interaction has antioxidant and anti-inflammatory properties in Parkinson’s disease animal model. Neuropharmacology.

[48] Kaur M., Aran K.R. (2025). Unraveling the role of Nrf2 in dopaminergic neurons: A review of oxidative stress and mitochondrial dysfunction in Parkinson’s disease. Metab. Brain Dis..

[49] Pajares M., Cuadrado A., Rojo A.I. (2017). Modulation of proteostasis by transcription factor NRF2 and impact in neurodegenerative diseases. Redox Biol..

[50] Bahn G., Park J.S., Yun U.J., Lee Y.J., Choi Y., Park J.S., Baek S.H., Choi B.Y., Cho Y.S., Kim H.K. (2019). NRF2/ARE pathway negatively regulates BACE1 expression and ameliorates cognitive deficits in mouse Alzheimer’s models. Proc. Natl. Acad. Sci. USA.

[51] Hiramatsu G., Mizutani R., Toume K., Inada Y., Sawahata M., Uta D., Komatsu K., Kume T. (2025). Preventive Effects of Psora-leae Semen Extracts on Cognitive Dysfunction in Alzheimer’s Disease Model App(NL-P-F) Mice. Biol. Pharm. Bull..

[52] Arthur R., Navik U., Kumar P. (2025). Artemisinin Ameliorates the Neurotoxic Effect of 3-Nitropropionic Acid: A Possible Involvement of the ERK/BDNF/Nrf2/HO-1 Signaling Pathway. Mol. Neurobiol..

[53] Tucci P., Lattanzi R., Severini C., Saso L. (2022). Nrf2 Pathway in Huntington’s Disease (HD): What Is Its Role?. Int. J. Mol. Sci..

[54] Yang B., Pan J., Zhang X.N., Wang H., He L., Rong X., Li X., Peng Y. (2023). NRF2 activation suppresses motor neuron ferroptosis induced by the SOD1(G93A) mutation and exerts neuroprotection in amyotrophic lateral sclerosis. Neurobiol. Dis..

[55] Ito T., Ohuchi K., Kurita H., Murakami T., Takizawa S., Fujimaki A., Murata J., Oida Y., Hozumi I., Kitaichi K. (2025). Activated Fibroblast Growth Factor Receptor 1 Mitigated Poly-PR-Induced Oxidative Stress and Protein Translational Impairment. Biol. Pharm. Bull..

[56] Vo A.T.T., Khan U., Liopo A.V., Mouli K., Olson K.R., McHugh E.A., Tour J.M., Pooparayil Manoj M., Derry P.J., Kent T.A. (2024). Harshly Oxidized Activated Charcoal Enhances Protein Persulfidation with Implications for Neurodegeneration as Exemplified by Friedreich’s Ataxia. Nanomaterials.

[57] Wu Y.L., Sun H.L., Chang J.C., Lin W.Y., Chen P.Y., Chen C.C., Lee L.Y., Li C.C., Hsieh M., Chen H.W. (2024). Erinacine A-Enriched Hericium erinaceus Mycelium Ethanol Extract Lessens Cellular Damage in Cell and Drosophila Models of Spinocerebellar Ataxia Type 3 by Improvement of Nrf2 Activation. Antioxidants.

[58] Zhou A., Feng H.Y., Fan C.N., Wang J., Yuan Z.Y., Xu G.H., Li C.F., Huang W.F., Yi L.T. (2025). Asiaticoside Attenuates Chronic Restraint Stress-Induced Hippocampal CA1 Neuronal Ferroptosis via Activating BDNF/Nrf2/GPX4 Signaling Pathway. Drug Des. Dev. Ther..

[59] Herbet M., Szumełda I., Piątkowska-Chmiel I., Gawrońska-Grzywacz M., Dudka J. (2021). Beneficial effects of combined admin-istration of fluoxetine and mitochondria-targeted antioxidant at in behavioural and molecular studies in mice model of de-pression. Behav. Brain Res..

[60] Shirai Y., Fujita Y., Hashimoto R., Ohi K., Yamamori H., Yasuda Y., Ishima T., Suganuma H., Ushida Y., Takeda M. (2015). Dietary Intake of Sulforaphane-Rich Broccoli Sprout Extracts during Juvenile and Adolescence Can Prevent Phencyclidine-Induced Cognitive Deficits at Adulthood. PLoS ONE.

[61] Peng Z., Jia Q., Mao J., Jiang S., Ma Q., Luo X., An Z., Huang A., Ma C., Yi Q. (2025). The role of ferroptosis and oxidative stress in cognitive deficits among chronic schizophrenia patients: A multicenter investigation. Schizophrenia.

[62] Sakuma R., Minato Y., Maeda S., Yagi H. (2025). Nrf2 phosphorylation contributes to acquisition of pericyte reprogramming via the PKCδ pathway. Neurobiol. Dis..

[63] Bink D.I., Ritz K., Mackaaij C., Stam O., Scheffer S., Mizee M.R., Ploegmakers H.J., van Het Hof B.J., de Boer O.J., Sluimer J.C. (2025). Lack of intracranial atherosclerosis in various atherosclerotic mouse models. Vasc. Biol..

[64] Fang J., Wang H., Zhou J., Dai W., Zhu Y., Zhou Y., Wang X., Zhou M. (2018). Baicalin provides neuroprotection in traumatic brain injury mice model through Akt/Nrf2 pathway. Drug Des. Dev. Ther..

[65] Shi G., Cao Y., Xu J., Chen B., Zhang X., Zhu Y., Liu L., Liu X., Zhang L., Zhou Y. (2025). Inhibition of S100A8/A9 ameliorates neuroinflammation by blocking NET formation following traumatic brain injury. Redox Biol..

[66] Sánchez-Sanz A., Coronado-Albi M.J., Muñoz-Viana R., García-Merino A., Sánchez-López A.J. (2024). Neuroprotective and Anti-Inflammatory Effects of Dimethyl Fumarate, Monomethyl Fumarate, and Cannabidiol in Neurons and Microglia. Int. J. Mol. Sci..

[67] El Ali Z., Ollivier A., Manin S., Rivard M., Motterlini R., Foresti R. (2020). Therapeutic effects of CO-releaser/Nrf2 activator hybrids (HYCOs) in the treatment of skin wound, psoriasis and multiple sclerosis. Redox Biol..

[68] Tebay L.E., Robertson H., Durant S.T., Vitale S.R., Penning T.M., Dinkova-Kostova A.T., Hayes J.D. (2015). Mechanisms of activation of the transcription factor Nrf2 by redox stressors, nutrient cues, and energy status and the pathways through which it attenuates degenerative disease. Free Radic. Biol. Med..

